# LC–MS/MS-based quantitative study of the acyl group- and site-selectivity of human sirtuins to acylated nucleosomes

**DOI:** 10.1038/s41598-018-21060-2

**Published:** 2018-02-08

**Authors:** Kana Tanabe, Jiaan Liu, Daiki Kato, Hitoshi Kurumizaka, Kenzo Yamatsugu, Motomu Kanai, Shigehiro A. Kawashima

**Affiliations:** 1 Graduate School of Pharmaceutical Sciences, The University of Tokyo, 7-3-1 Hongo, Bunkyo-ku, Tokyo, 113–0033 Japan; 2 JST-ERATO, Kanai Life Science Catalysis Project, 7-3-1 Hongo, Bunkyo-ku, Tokyo, 113–0033 Japan; 3 Laboratory of Structural Biology, Graduate School of Advanced Science and Engineering, Waseda University, 2–2 Wakamatsu-cho, Shinjuku-ku, Tokyo, 162–8480 Japan

## Abstract

Chromatin structure and gene expression are dynamically regulated by posttranslational modifications of histones. Recent advance in mass spectrometry has identified novel types of lysine acylations, such as butyrylation and malonylation, whose functions and regulations are likely different from those of acetylation. Sirtuins, nicotinamide adenine dinucleotide (NAD^+^)-dependent histone deacetylases, catalyze various deacylations. However, it is poorly understood how distinct sirtuins regulate the histone acylation states of nucleosomes that have many lysine residues. Here, we provide mass spectrometry-based quantitative information about the acyl group- and site-selectivity of all human sirtuins on acylated nucleosomes. The acyl group- and site-selectivity of each sirtuin is unique to its subtype. Sirt5 exclusively removes negatively-charged acyl groups, while Sirt1/2/3/6/7 preferentially remove hydrophobic acyl groups; Sirt1 and Sirt3 selectively remove acetyl group more than butyryl group, whereas Sirt2 and Sirt6 showed the opposite selectivity. Investigating site-selectivity for active sirtuins revealed acylated lysines on H4 tails to be poor substrates and acylated H3K18 to be a good substrate. Furthermore, we found Sirt7 to be a robust deacylase of H3K36/37, and its activity reliant on nucleosome-binding at its *C*-terminal basic region. All together, our quantitative dataset provides a useful resource in understanding chromatin regulations by histone acylations.

## Introduction

The structure of chromatin, a polymer of nucleosomes, is dynamically regulated by post-translational modifications (PTMs) of the histones in nucleosomes. Histone acetylation is one of the most prominent regulatory PTMs in epigenetics, and is often a key in activating gene expression^1^. Cationic lysine residues lose their charge upon acetylation, promoting chromatin decompaction and gene transcription. In addition, histone acetylation can indirectly promote transcription through the intermediary “Readers” of acetylated lysines, such as the bromodomain proteins^2^. Histones are made of two domains differing in both structure and function; the tail is unstructured and flexible, and the histone-fold forms the structural support of the nucleosome. One nucleosome contains over 100 lysine residues, with many in the tail region being heavily acetylated in living cells. The acetylation level of each lysine residue of the histones is determined by the balance between two types of enzymes: histone acetyltransferases (HATs) and histone deacetylases (HDACs). However, we lack information about lysine residue selectivity in the nucleosomes of these enzymes.

HDACs can be classified into two major groups: zinc-dependent HDACs (HDAC1–11) and nicotinamide adenine dinucleotide (NAD^+^)-dependent ones^3^. The latter, called sirtuins, are known to regulate important physiologic functions, such as metabolism, cell cycle, genome maintenance, and aging processes^4–8^. They are thus considered attractive medicinal targets for the metabolic and aging-related diseases^9^. Sirtuins have seven isoforms in humans, Sirt1–7, whose deacetylase activity and cellular localization differ widely from each other, although their family of proteins share a catalytic domain that is conserved among all sirtuins. Using an acetylome peptide microarray, the research has revealed that different isoforms of human sirtuins have different substrate sequence preferences^10^. Although there are several studies examining the site-selectivity of sirtuins towards histones, most studies have used non-nucleosomal substrates, such as histone-tail peptides or free histones^11–14^. However, recent studies have suggested that sirtuin reactivity and the site selectivity may differ between nucleosomal and non-nucleosomal substrates^15,16^. Furthermore, quantitative information is limited, as most research has used western blotting with anti-acetyl lysine antibodies to evaluate substrate lysine site-selectivity.

Recent progresses in mass spectrometry have identified novel types of lysine acylations on histones^17,18^. For example, butyrylation introduces a two-carbon longer, more hydrophobic acyl group (i.e., butyryl group) than acetylation. Recent studies have revealed that histone butyrylation could be catalyzed by the same HATs that promote histone acetylation^19^. In addition, butyrylation of histones activates transcription from a chromatin template in the same manner as acetylation, suggesting that the effects of butyrylation on chromatin structure *in vitro* may be similar to those of acetylation. Acylations introducing acidic acyl groups, however, can change the charge state of the lysine residue from cationic to anionic. For example, malonylation introduces an acyl group with a similar molecular size to butyrylation, but the malonyl group has an anionic carboxylate group. Our biochemical analyses using recombinant nucleosomes that are acetylated or malonylated by artificial catalyst systems suggest that histone malonylation has a greater effect on inter-nucleosome interactions than acetylation does^20,21^, while the functions of acidic acyl groups on histones in living cells remain to be elucidated. Enzymes that catalyze the removal of acyl groups other than the acetyl group have been only partly identified thus far. Sirt1–3 can remove hydrophobic acyl groups^22,23^, while Sirt5 removes acidic acyl groups^24–26^. It has been shown that, using model peptides, Sirt6 efficiently removes long-chain acyl groups^27–29^, and that Sirt4 removes acidic acyl groups, such as methylglutaryl, hydroxymethylglutaryl, and 3-methylglutaconyl ones^30^. Site-selectivity of HDAC isoforms on histones bearing non-acetyl acylations also remains to be examined. Herein we report on characterization of the acyl group- and site-selectivity of seven human sirtuins using acylated nucleosomes as substrates, by quantitative mass spectrometry-based analysis.

## Results

### LC–MS/MS-based quantitative analyses for site- and acyl group-selectivity of human sirtuins on acylated nucleosomes

**Figure 1:**
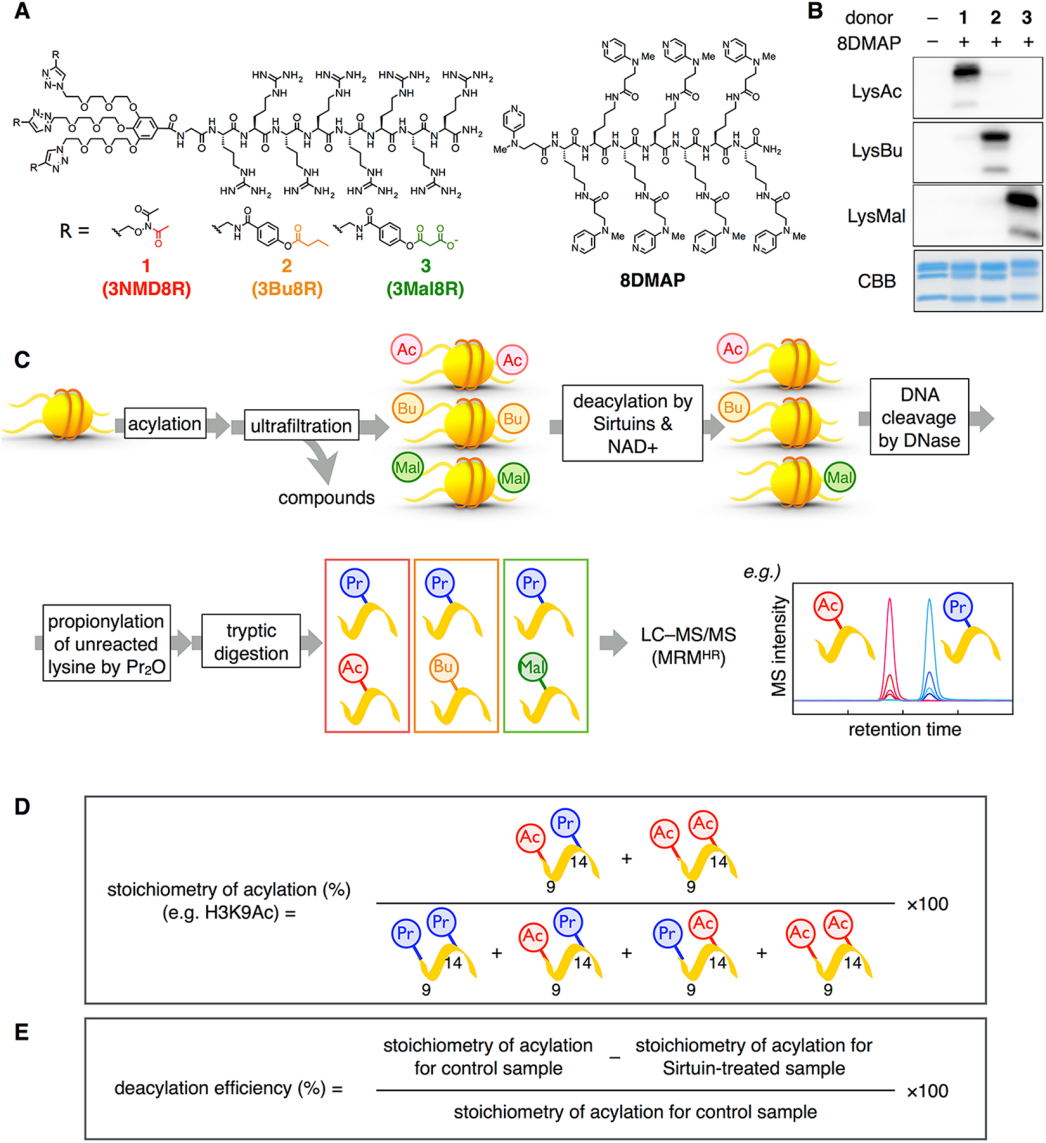
Methods to quantitatively analyze the selectivity of sirtuins. (**A**) Chemical structures of acyl donors **1**–**3** and catalyst (**8DMAP**). (**B**) Western blotting of nucleosomes acylated by **1–3** and **8DMAP**, detected with an anti-Ac-Lys, an anti-Bu-Lys, or an anti-Mal-Lys antibody. Uncropped images are shown in Fig. S5. (**C**) Schematic experimental strategy for HDAC assay to study site- and acyl group-selectivity of sirtuins. First, acylated lysines are used as substrates for the HDAC assays. After cleavage of DNA, propionylation of unreacted lysine is conducted. Then, tryptic digestion is carried out to obtain tryptic peptides, which are allowed to quantitatively analyze using LC–MS/MS. (**D**) Calculation method for the stoichiometry of acylated lysines. For example, the stoichiometry of acetylation on H3K9, which was detected in the peptide H3[9–17] together with K14, was calculated by dividing the sum of the peak areas of K9AcK14Pr and K9AcK14Ac by the sum of the peak areas of K9PrK14Pr, K9AcK14Pr, K9PrK14Ac and K9AcK14Ac. (**E**) Calculation method for deacylation efficiency, which describes the conversion from acylated lysine into deacylated one.

**Figure 2:**
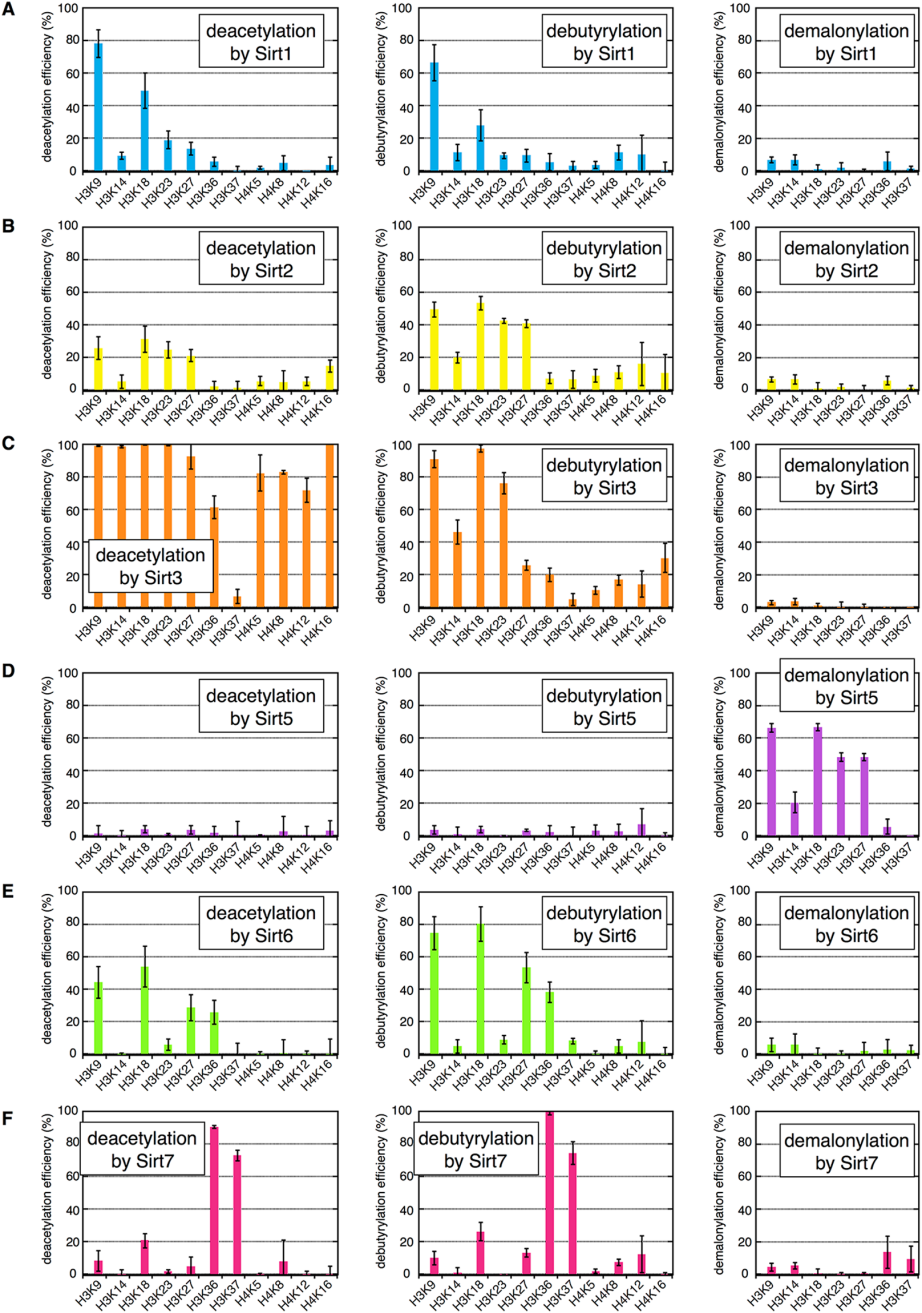
Results for HDAC assay. (**A**) deacetylation (left), debutyrylation (middle), and demalonylation (right) efficiencies on H3K9/14/18/23/27/36/37 and H4K5/8/12/16 of Sirt1 (24 ng/µL). (**B**) Those of Sirt2 (24 ng/µL). (**C**) Those of Sirt3 (12 ng/µL). (**D**) Those of Sirt5 (2 ng/µL). (**E**) Those of Sirt6 (12 ng/µL). (**F**) Those of Sirt7 (12 ng/µL). The average and SD (error bars) obtained from *n* = 3 independent HDAC assays are indicated, where measurements were conducted in duplicate for each HDAC assay.

We have previously developed artificial catalyst systems composed of a nucleosome-binding catalyst (8DMAP) and acyl donors, including an acetyl donor (3NMD8R, **1**) and a malonyl donor (3Mal8R, **3**)^20^ (Fig. 1A). Using these artificial catalyst systems, lysine residues of histone tails in recombinant nucleosomes could be easily acetylated or malonylated in a similar residue-pattern to HATs. Therefore, we envisioned that acetylated or malonylated nucleosomes could be used as substrates for HDAC-promoted deacylation to compare the site- and acyl group-selectivity of HDAC isoforms. In addition, to expand the repertoire of the artificial acylation system to butyrylation, a butyryl donor (3Bu8R, **2**) was prepared (Fig. 1A), which has the same backbone structure to the malonyl donor (**3**). By using those compounds, acetylated, butyrylated, or malonylated nucleosomes were prepared, as confirmed by western blotting using anti-acetyl lysine, anti-butyryl lysine, or anti-malonyl lysine antibody, respectively (Fig. 1B). After the small molecules were removed by ultrafiltration, acylated nucleosomes were used as substrates for the subsequent deacylation assay (Fig. 1C). We conducted HDAC assay using recombinant sirtuins, Sirt1–7, against acetylated, butyrylated, and malonylated nucleosomes (Fig. S1). After *in vitro* HDAC assay was carried out, propionylation of unmodified lysines, tryptic digestion, and quantitative liquid chromatography – tandem mass spectrometry (LC–MS/MS) analyses were subsequently conducted (Fig. 1C). The stoichiometry of acylation was calculated in a similar manner to the previous reports^31–34^ (Fig. 1D, Fig. S2). The efficiency of deacylation was quantitatively evaluated by the division of the difference between the stoichiometries of acylation in control and sirtuin-treated samples, by that of control samples. This parameter denotes the conversion efficiency from acylated lysine residues into unmodified ones (Fig. 1E, Fig. S3). The resulting deacylation efficiencies by each sirtuin are shown in Fig. 2, except for Sirt4, which did not show any detectable deacylase activity in our experiments (Fig. S4).

### Acyl group-selectivity of sirtuins

**Figure 3:**
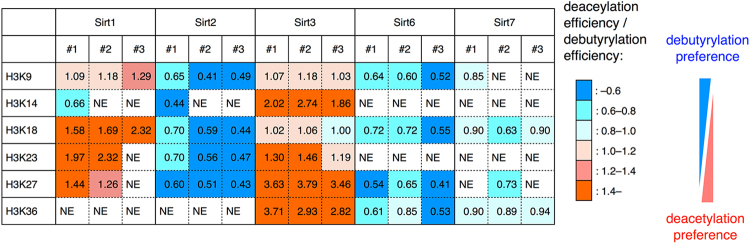
Acyl group-selectivity of sirtuins. The value in each square is “acetyl preference index (API)”, obtained by dividing deacetylation efficiency by debutyrylation efficiency. NE: not estimated because deacetylation and debutyrylation efficiencies were less than 10%, and thus, were not reliable. Red indicates high preferences for deacetylation, and blue indicates high preferences for debutyrylation. #1, #2, and #3 are three independent HDAC assays.

Only Sirt5 has remarkable demalonylaton activity (Fig. 2D) as reported previously^25^. On the other hand, other sirtuins had both deacetylase and debutyrylase activity (Fig. 2A–F). We analyzed the preferences of each sirtuin for acetyl versus butyryl groups, except for Sirt5 which did not show detectable deacetylation/debutyrylation activity. “Acetyl preference index (API)” was calculated at each lysine on H3, which was defined by the ratio between deacetylation and debutyrylation efficiencies (Fig. 3). APIs of Sirt1 at H3K18 were 1.58, 1.69, and 2.32 in three independent experiments, which means that Sirt1 prefers deacetylation compared to debutyrylation at this site. Sirt1 showed similar tendency at other lysine residues, indicating high acetyl group preferences of Sirt1 throughout the lysine residues tested. Similarly, it was revealed that Sirt3 has high acetyl group preferences. On the other hand, Sirt2 and Sirt6 showed obvious butyryl group preferences, where APIs ranged from 0.41 to 0.72 throughout the H3 tails. In addition, APIs of Sirt7 were 0.63–0.94, suggesting slight debutyrylation preferences of Sirt7. Therefore, we concluded that Sirt1 and Sirt3 prefer deacetylation to debutyrylation, whereas Sirt2, Sirt6, and Sirt7 have the opposite preferences.

### Site-selectivity of sirtuins

**Figure 4:**
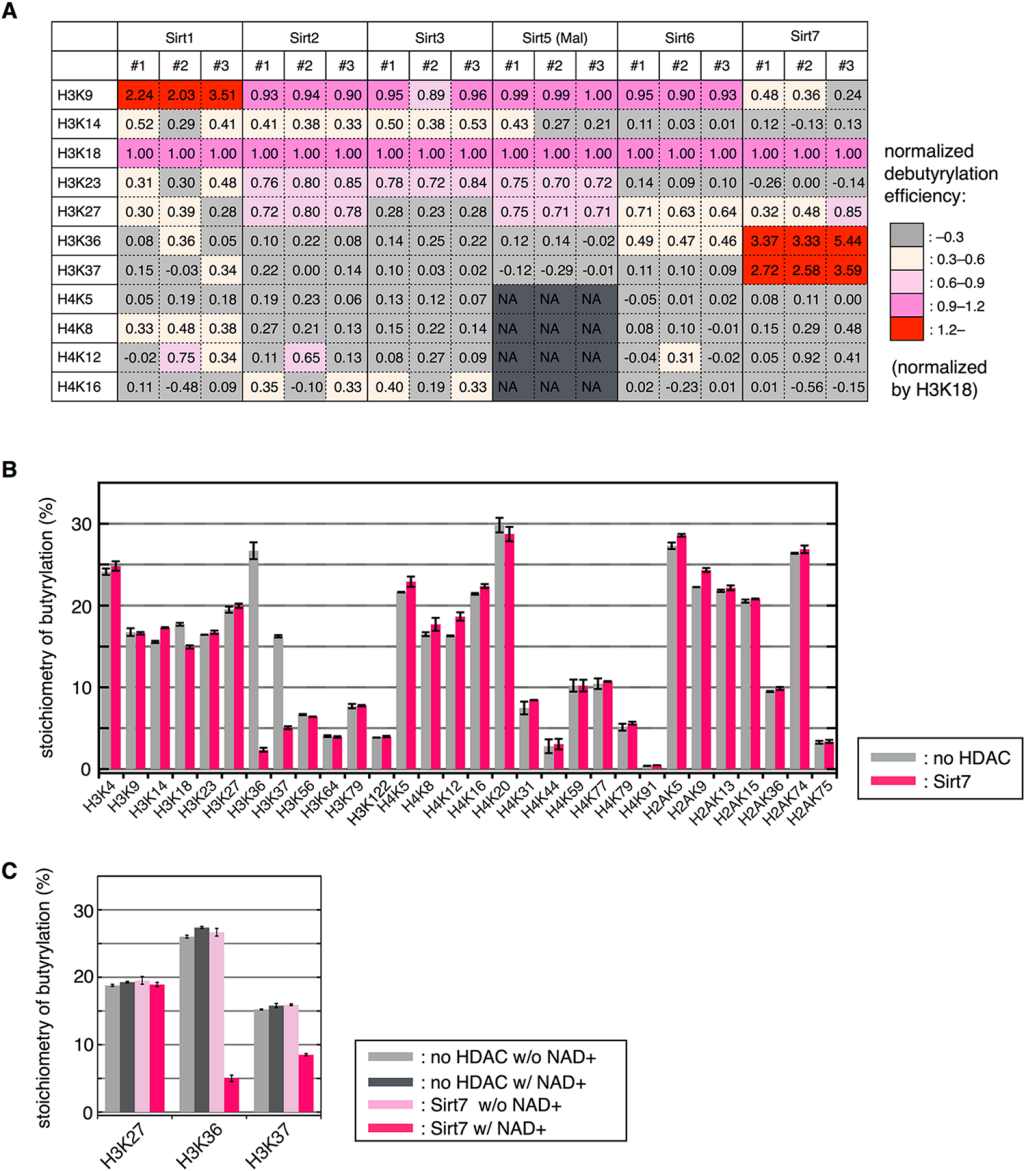
Site-selectivity of sirtuins. (**A**) Debutyrylation efficiency normalized by that at H3K18. In the case of Sirt5, demalonylation efficiency was adopted, and lysine residues on H4 tail were not analyzed (NA). Red cells indicate that the butyryl group at the corresponding lysine was more efficiently removed by sirtuins than that at H3K18. #1, #2, and #3 are three independent HDAC assays. (**B**) Comprehensive evaluation of site-selectivity of Sirt7 at lysine residues on H3, H4, and H2A. Gray columns: stoichiometry of butyrylation in control sample; Pink columns: that in Sirt7-treated sample. (**C**) NAD^+^-dependent debutyrylation of H3K36/K37 by Sirt7. The chart shows the stoichiometry of butyrylation after HDAC assay in the presence or in the absence of Sirt7 and/or NAD^+^.

The deacylase activity of sirtuins depends on the position of the lysine residue (Fig. 2). Intriguingly, we found that several lysine residues on H3 tails were much more efficiently deacylated by all the sirtuins than those on H4 tails (Fig. 2). In order to study site-selectivity of sirtuins within H3 tails, each deacylation efficiency was normalized by the corresponding deacylation at H3K18 (Fig. 2). Normalized debutyrylation efficiencies of Sirt1–3, Sirt6, and Sirt7 and normalized demalonylation efficiencies of Sirt5 are shown in Fig. 4A. For example, normalized debutyrylation efficiencies of Sirt1 at H3K9 was 2.24, 2.03, and 3.51 in three independent experiments, which suggests that the butyryl group at H3K9 was more susceptible to debutyrylation by Sirt1, than the butyryl group at H3K18. In addition, those of Sirt2, Sirt3, Sirt5, and Sirt6 at H3K9 were around 0.89–1.00, indicating that the butyryl group at H3K9 was as efficiently removed by those sirtuins as that at H3K18. In sharp contrast, normalized debutyrylation efficiencies at H3K9 by Sirt7 were 0.24–0.48, suggesting that Sirt7 did not efficiently debutyrylate H3K9 compared to H3K18, which is consistent to the previous report showing Sirt7 as a deacetylase for H3K18^35^. Intriguingly, it was found that normalized debutyrylation efficiencies at H3K36 and K37 were 3.33–5.44 and 2.58–3.59, respectively, suggesting that the butyryl groups at H3K36 and K37 are highly efficiently removed by Sirt7 compared to the butyryl group at H3K18. To address this site selectivity of Sirt7 in more detail, debutyrylation efficiency at other lysine residues was also examined (Fig. 4B). Although the stoichiometry of butyrylation at 30 lysine residues on H3, H4, and H2A was quantified in similar manner to H3 and H4 tails, only H3K36 and K37 were significantly debutyrylated, with only a slight debutyrylation at H3K18. No detectable debutyrylation was observed at other sites (Fig. 4B). These findings, together with the facts that this debutyrylase activity is NAD^+^-dependent (Fig. 4C) and the site-selectivity of deacetylation was similar to debutyrylation (Fig. 2F), led to the conclusion that Sirt7 selectively deacetylates and debutyrylates H3K36/K37 more efficiently than H3K18.

### Analysis of H3K36/K37-selectivity of Sirt7

**Figure 5:**
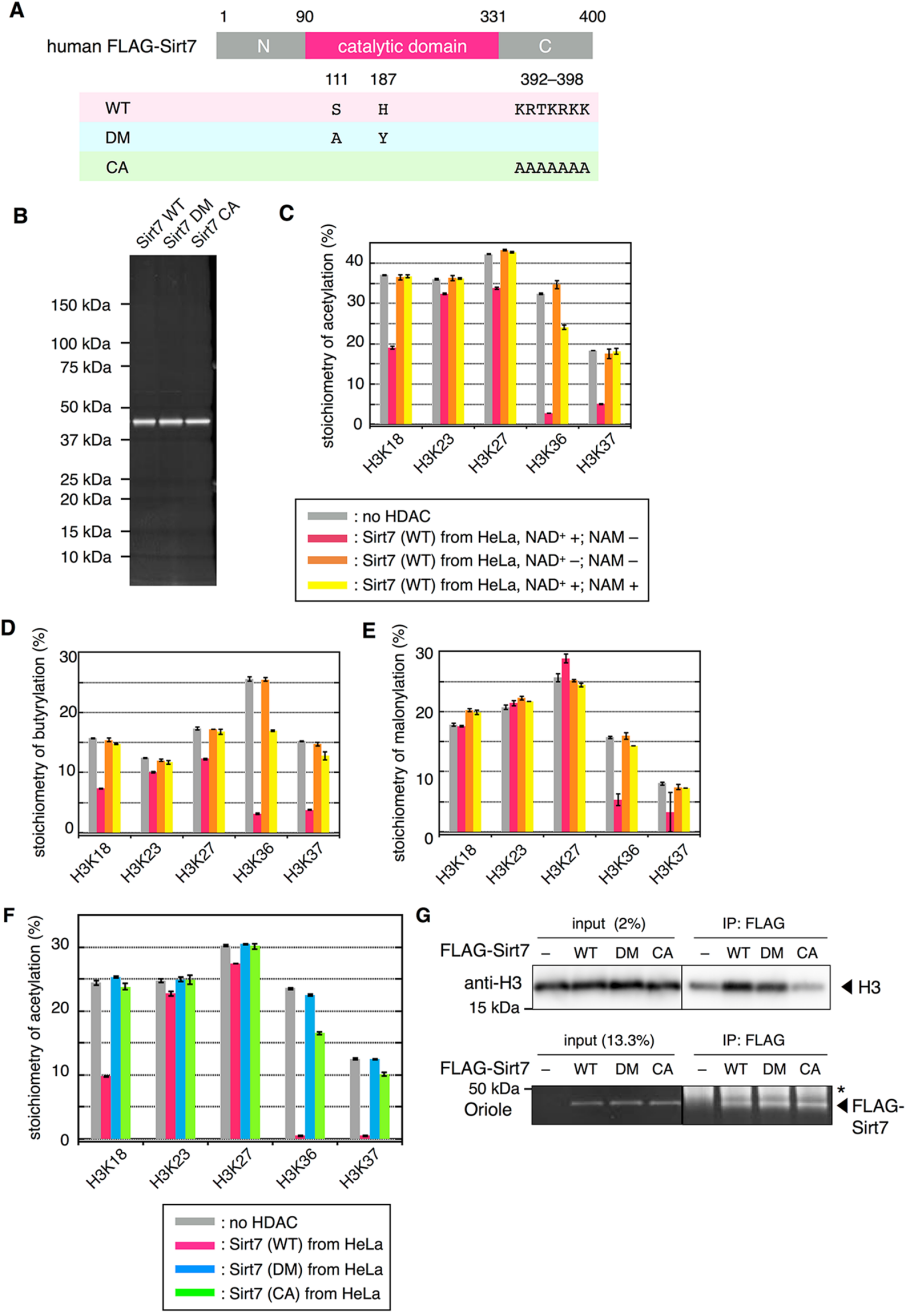
Analysis of H3K36/K37 selectivity by Sirt7 using FLAG-Sirt7 mutants purified from HeLa cells. (**A**) Schematics showing the mutated residues in the two FLAG-Sirt7 mutants. WT: wild type; DM: double mutant (S111A/H187Y); CA: mutant where *C*-terminal basic region has mutated to seven alanine residues. (**B**) SDS-PAGE analysis of purified FLAG-Sirt7 mutants. Proteins were visualized by Oriole stain. (**C**) Deacetylation assay using purified FLAG-Sirt7 (WT, 2.0 ng/µL) in the presence or absence of NAD^+^ (2 mM) and/or NAM (10 mM). Gray: control sample; pink: Sirt7-treated sample with NAD^+^; orange: Sirt7-treated sample without NAD^+^; yellow: Sirt7-treated sample with NAD^+^ and NAM. Error bars indicate upper and lower limits of two independent measurements. (**D**) Debutyrylation assay using purified FLAG-Sirt7 (WT, 2.0 ng/µL) in the presence or absence of NAD^+^ (2 mM) and/or NAM (10 mM). Colors of the columns correspond to samples described in (**C**). Error bars indicate upper and lower limits of two independent measurements. (**E**) Demalonylation assay using purified FLAG-Sirt7 (WT, 3.6 ng/µL) in the presence or absence of NAD^+^ (2 mM) and/or NAM (10 mM). Colors of the columns correspond to samples described in (**C**). Error bars indicate upper and lower limits of two independent measurements. (**F**) Deacetylation assay using FLAG-Sirt7 mutants purified from HeLa cells (5 ng/µL). The graph shows the stoichiometry of acetylation after HDAC assay. Gray: control sample without Sirt7; pink: Sirt7 (WT)-treated sample; blue: Sirt7 (DM)-treated sample; green: Sirt7 (CA)-treated sample. Error bars indicate upper and lower limits of two independent measurements. (**G**) Immunoblot of recombinant nucleosomes on their own (input) or bound to beads containing the indicated FLAG-Sirt7 proteins using anti-histone H3 antibody. The bottom panel shows input or bead-bound FLAG-Sirt7 stained with Oriole. Asterisk indicates heavy chains of anti-FLAG antibody. The grouping of images from different parts of the same gel is indicated by dividing lines. Uncropped images of (**B**) and (**G**) are shown in Fig. S6.

Previously documented activity of Sirt7 for histones *in vitro* is controversial. Denu’s group reported on acyl group-selectivity of seven human sirtuins using acylated H3K9 peptides, where no appreciable amounts of deacylated peptides were observed with Sirt7^29^. Chua and coworkers have reported that Sirt7 showed highly specific deacetylase activity on acetylated H3K18 peptide, and that Sirt7 also showed robust and specific H3K18Ac-deacetylase activity on full-length histone H3 in purified poly-nucleosomes^35^. Recently, Lin’s group reported activation of deacylase activity of Sirt7 by double-stranded DNA (dsDNA) and RNA^36,37^. In the absence of dsDNA, no deacetylation was observed on either of acetylated H3K9 and H3K18 peptides or purified histones, whereas it displayed deacetylase activity on both peptides and purified histones in the presence of dsDNA. In addition, they have reported that Sirt7 showed robust deacetylase activity against H3K18 on the nucleosomes. They also reported that RNA enhanced the activity of Sirt7 against acetylated H3K18 peptide. RNA binding motif of Sirt7 resides in its *C*-terminal basic region, since Sirt7 mutant (CA; Fig. 5A), in which the *C*-terminal basic amino acids were mutated to seven alanine residues (K392A/R393A/T394A/K395A/R396A/K397A/K398A), exhibited reduced RNA-binding abilities and was unable to be activated by the addition of RNAs^37^. Our results showed that Sirt7 has robust deacetylase or debutyrylase activity on acetylated or butyrylated nucleosomes, mainly toward H3K36 and K37, without the addition of RNAs (Fig. 4). Therefore, we hypothesized that Sirt7 might bind with DNA on nucleosomes via *C*-terminal basic region, and its nucleosome-binding would enhance deacylase activity of Sirt7. In order to address this hypothesis, we purified the FLAG-Sirt7 (CA) mutant protein as well as the wild-type FLAG-Sirt7 (WT) and the catalytic dead FLAG-Sirt7 (DM) mutant proteins from HeLa cells (Fig. 5A,B). The purified FLAG-Sirt7 (WT) protein showed strong deacetylase and debutyrylase activity toward H3K36/K37, and also weakly deacetylated or debutyrylated K18/23/27 (Fig. 5C,D). It should be noted that the purified FLAG-Sirt7 (WT) protein showed higher activity than commercially available recombinant Sirt7 (Fig. S1), since we used smaller amount of purified FLAG-Sirt7 (WT) (2 ng/µL in Fig. 5C) than recombinant one (12 ng/µL in Figs 2–4). Furthermore, the FLAG-Sirt7 (WT) removed the malonyl group specifically at H3K36/K37, although its demalonylase activity was weaker than deacetylation or debutyrylation one (Fig. 5E). All the observed deacylase activity was dependent on the presence of NAD^+^, and significantly inhibited by the addition of nicotinamide (NAM), which has been shown to inhibit Sirt7 activity *in vitro*^35^ (Fig. 5C–E). In addition, the FLAG-Sirt7 (DM) mutant protein completely lost its deacylase activity (Fig. 5F). These results indicate that observed deacylations were catalyzed by FLAG-Sirt7 protein itself, but not other factors that might be co-purified with Sirt7. Pull-down assay showed that Sirt7 (WT) and Sirt7 (DM), but not Sirt7 (CA), bound with recombinant nucleosomes (Fig. 5G), suggesting that Sirt7 directly interacts with nucleosomes via the C-terminal basic region. Sirt7 (CA) mutant showed significantly reduced deacylation activity as compared to Sirt7 (WT) (Fig. 5F), which supports our hypothesis that Sirt7 binds with DNA on nucleosomes *via C*-terminal basic region, and its nucleosome-binding enhances deacylase activity of Sirt7 toward H3K36/37.

## Discussion

In this study, we have provided LC–MS/MS-based quantitative information about site- and acyl group-selectivity of all the human sirtuins against acetylated, butyrylated, and malonylated nucleosomal substrates and found that site- and acyl-group selectivity of each sirtuin are different from each other. Regarding site-selectivity, we found two common features among sirtuins; (1) acylated lysine residues on H4 tails are poor substrates, (2) acylated H3K18s are generally susceptible to deacylation. These findings are not completely consistent with the previous studies using non-nucleosomal substrates. For example, when human Sirt1 was incubated with acetylated human core histones purified from HeLa cells, the acetyl group at H4K16 was more efficiently removed by Sirt1 than that at H3K9^11^. Our data suggests that the nucleosome structure may limit accessibility of sirtuins to H4. One possibility would be that all sirtuins may similarly bind to nucleosomes, probably through their conserved catalytic domain, and thus H3 tails, but not H4 tails, would be located near the catalytic pockets of all the sirtuins. We also found unique site-selectivity of Sirt1 and Sirt7. Sirt1 preferentially removes H3K9 acylations. This result is consistent with the fact that the amino acid sequence around H3K9 completely matches to the high-affinity sequence of Sirt1, revealed by an acetylome peptide microarray^10^. In contrast, our finding that Sirt7 removed acetyl and butyryl groups from H3K36/37 more efficiently than from H3K18Ac on nucleosomes was unexpected, since it has been reported that Sirt7 is a highly selective H3K18Ac deacetylase^35^. In the previous study, a series of acetylated histone peptides were used as substrates of Sirt7 *in vitro*, but histone peptides with H3K36Ac or H3K37Ac were not examined^35^. Our data indicate that nucleosome-binding of Sirt7 through its *C*-terminal basic region is critical for efficient H3K36/37 deacylations on nucleosome. The polybasic amino acids (residue 392–398, KRTKRKK) of Sirt7 are also essential for Sirt7 localization in nucleolus, where ribosomal RNAs are enriched^38^. Therefore, Sirt7 activity might be spatially regulated by distinct nucleic acids in cells. Nucleosomal DNAs could activate Sirt7 on chromatin, whereas ribosome RNAs could activate Sirt7 in nucleolus. Addressing whether deacylation on H3K36/37 by Sirt7 is physiologically relevant would be an important next step. Since mono-, di-, and tri-methylation at H3K36 are important epigenetic marks for chromatin structure and functions^39^, one hypothesis to be examined states that Sirt7-mediated deacylations at H3K36/37 may be prerequisite for H3K36 methylation that should be regulated in a spatiotemporal manner.

Regulation of various acylation states in histones is one of the important questions to understand chromatin epigenetics. Our quantitative analyses show that: (1) while Sirt1, 2, 3, 6, and 7 preferentially remove hydrophobic acyl groups over the malonyl group (acyl group-selectivity between hydrophobic *versus* anionic acyl groups); (2) Sirt1 and Sirt3 preferentially remove the acetyl group over the butyryl group, whereas Sirt2 and Sirt6 preferentially remove the butyryl group over the acetyl group (acyl group-selectivity between acetyl *versus* butyryl group); (3) Sirt5 selectively removes negatively-charged malonyl group compared to hydrophobic ones; and (4) Sirt4 did not show any detectable deacylase activity in our experiments. Interestingly, previous study using mono-acylated peptide substrates showed that not only Sirt1 and Sirt3, but also Sirt2 removed the acetyl group more efficiently than the butyryl group^28^. Therefore, the acyl group-preferences of Sirt2 may be different depending on whether binding to nucleosomes or to peptides. Analysis of co-crystal structure of Sirt2 and nucleosomes may be informative to address this hypothesis. The butyryl group-preference of Sirt6 is consistent with the previous kinetic analysis, in which catalytic efficiency (*k*_cat_/*K*_m_) of Sirt6 on H3K9-butyrylated peptides (10 s^−1^M^−1^) was about twice as much as that on H3K9-acetylared peptides (4.8 s^−1^M^−1^)^40^. The ratio of the amount of acetylated *versus* butyrylated histones could be affected by the balance between acetyl group-preferred sirtuins and butyryl group-preferred sirtuins. A recent study has shown that lysine butyrylation on histones is enriched in the later steps of spermatogenesis compared to lysine acetylation^19^. It would be interesting to examine whether the localization or protein level of each sirtuin changes during spermatogenesis.

Besides hydrophobic and acidic acylations, hydroxylated acyl groups including β-hydroxybutyryl and 2-hydroxyisobutyryl modifications have been recently identified^41,42^. The hydroxy group in such acyl groups can form hydrogen bonds with other molecules. We anticipate that sirtuins’ selectivity toward hydroxylated acyl groups can be addressed by identifying a method to synthesize the nucleosomes furnishing such acyl groups. Our previously developed artificial catalyst system, however, cannot adopt such acyl groups at this stage. New methods are required to address this issue. Furthermore, our system can be also applied to understand acyl group-selectivity of Zn^2+^-dependent HDACs (HDAC1–11), whose selectivity has been rarely studied, except for HDAC3 that showed weak decrotonylase activity^43^. We conclude that our LC–MS/MS-based quantitative analyses for acyl group- and site-selectivity of HDAC isoforms on acylated nucleosomes are powerful tools for understanding how histone acylations are regulated, which enables to gain new insights into the fundamental epigenetic mechanisms.

## Methods

### Preparation of acylated nucleosomes

Nucleosomes were reconstituted and purified as described previously^20^. To a solution of recombinant nucleosomes (0.346 µM for DNA concentration) in 20 mM Tris-HCl (pH 7.5) buffer, 8DMAP and an acyl donor (**1**, **2**, or **3**) were sequentially added. The concentrations of the compounds are as follows: 8DMAP (10 µM) and acetyl donor **1** (5 µM) for acetylation; 8DMAP (5 µM) and butyryl donor **2** (2 µM) for butyrylation; 8DMAP (10 µM) and malonyl donor **3** (20 µM) for malonylation. After incubation at 25 °C for 3 h, compounds were removed by ultrafiltration using an Amicon Ultra-0.5 30 K device (Merck Millipore, cat. #UFC503024) as follows: First, ultrafiltration was conducted with 500 µL of TCST-EDTA (20 mM Tris-HCl (pH 7.5) buffer containing 1 mM DTT and 0.005% Triton X-100) in the presence of 300 mM NaCl four times, then it was conducted with 500 µL of TCST-EDTA four times, to remove NaCl. Western blotting using an anti-Ac-Lys antibody (Cell Signaling Technology cat. #9441), anti-butyrylated lysine antibody (PTM Biolabs, cat. #PTM-301) or anti-malonylated lysine antibody (PTM Biolabs, cat. #PTM-901) was conducted to confirm that nucleosomes were acylated.

### HDAC assay

*In vitro* deacylation assays were performed as described previously^20^ with some modifications. Acylated nucleosomes (1 µg/reaction) were incubated at 30 °C for 3 h with 24 ng/µL of recombinant Sirt1 (Active Motif, cat. # 31340), 24 ng/µL of recombinant Sirt2 (MyBiosource, cat. #MBS203851), 12 ng/µL of recombinant Sirt3 (Cyclex, cat. #CY-E1153), 3 ng/µL of recombinant Sirt4 (described below), 2 ng/µL of recombinant Sirt5 (Cyclex, cat. #CY-E1155), 12 ng/µL recombinant Sirt6 (Cyclex, cat. # CY-E1156), 12 ng/µL of recombinant Sirt7 (Sigma, cat. #SRP5274), or Flag-Sirt7 (described below) in a deacetylation buffer (2 mM NAD^+^, 25 mM Tris–HCl pH 8.0, 137 mM NaCl, 2.7 mM KCl, 1 mM MgCl_2_, and 25 µM ZnSO_4_) in a total volume of 25 µL. After TCA precipitation, the pellet was washed with ice-cold acetone (200 µL) twice, dried, and dissolved in 49.5 µL of DNase buffer (40 mM Tris–HCl pH 7.5, 8 mM MgCl_2_, and 5 mM DTT). After incubation with DNase I (Takara, cat. # 2270 A) for 30 min at 37 °C, histones were precipitated by adding ice-cold acetone (200 µL). The pellet was washed with acetone (200 µL) twice, dried, and dissolved into water (10 µL). To this solution, 10 µL of 200 mM NH_4_HCO_3_ aq., 20 µL of MeOH/propionic anhydride (3/1), and 18 µL of 28% ammonia solution were added stepwise. After incubation at room temperature for 30 min, the solvents were removed by a Speed-Vac evaporator. The pellet was dissolved in 10 µL of 0.1% protease MAX (Promega, cat. #V2072) in 50 mM NH_4_HCO_3_ aq. To this solution, 40 µL of 50 mM NH_4_HCO_3_ aq., 1 µg trypsin gold (Promega, cat. #V5280), and 0.5 µL of 1% protease MAX were added stepwise, and the mixture was incubated at 37 °C for 3 h. After adding 5% formic acid (25 µL), the solvent was removed by a Speed-Vac evaporator. The dried sample was dissolved into 0.1% formic acid (18 µL) and centrifuged (15,000 rpm, 10 min). The supernatant was used for LC–MS/MS analysis. 5 times diluted samples were used for analysis of H3K18 and H3K23.

### LC–MS/MS analysis and quantification of the stoichiometry of acylation

LC–MS/MS analyses were conducted as described previously^20^. Targeted precursor ions and collision energies are described in Tables S2. Measurements were conducted in duplicate for each HDAC assays. Data analysis was carried out using PeakView software (AB Sciex, version 1.2.0.3) as described previously^20^, and the average values obtained from duplicated measurements were adopted as the results for each HDAC assay. The stoichiometry of acylated lysines was calculated as a percentage of the total peak area of the extracted ion chromatogram for acetylated peptides in the sum of those for acylated peptides and propionylated peptides. Selected fragment ions are also shown in Tables S2. In the case of malonylation, malonylated fragment ions were extracted as decarboxylated forms. As ionization efficiency between malonylated and propionylated peptides are different, the peak areas of malonylated peptides were converted into those of propionylated peptides using authentic peptides as previously described^20^ (Table S3), except for the peptide containing H3K27, K36, and K37, where the ionization correction could not be conducted because there are three lysine residues on the peptide.

### Plasmids

Human Sirt7 cDNA was amplified by PCR from pcDNA3-hSirt7, which was kindly provided from Ass. Prof. Dr. Kimura (Tsukuba Univ.), and ligated into pEBB Flag GCN5 (Addgene, cat. #74784) after removal of the Gcn5 part to generate Flag-Sirt7 (WT). Sirt7 mutants including DM (S111A/H187Y) and CA (K392A/R393A/T394A/K395A/R396A/K397A/K398A) were generated by using PrimeSTAR Mutagenesis (Takara, cat. #R046A). The plasmid for expressing GST-Sirt4 was prepared as follows: a cDNA encoding residues 29–314 of human Sirt4 was cloned into the BamHI and SalI sites of the pGEX 6p-1 vector to obtain the corresponding construct.

### Preparation of recombinant Sirt4

GST-Sirt4 was co-expressed with GroEL/GroES/Tig in chaperone component cells pG-Tf2/BL21 (Takara, #9120). The resulting fusion protein was purified using Glutathione Sepharose 4B (GE Healthcare, #17–0756–01). Site-specific proteolysis of the fusion protein with PreScission Protease (GE Healthcare, #270843) was conducted for separation of the Sirt4 protein from the GST tag. After further purification by anion exchange column, co-expressed GroEL could not be completely removed, so we used the complex of Sirt4 and GroEL as it was.

### Cell culture, transfection and purification of Sirt7

HeLa cells were grown in Dulbecco’s modified Eagle’s medium with 10% FBS, GlutaMAX (1×), 100 U/mL penicillin, and 100 µg/mL streptomycin. Transfection was carried out with Lipofectamine LTX Reagent with Plus Reagent (Invitrogen, cat. #15338100) according to the manufacturer’s instructions. After 48 h, FLAG-tagged Sirt7 (WT) and the corresponding mutants (DM, CA) were collected and lysed in lysis buffer (50 mM Tris-HCl (pH 7.5), 300 mM NaCl, 1% Nonidet P-40, 1 mM EDTA, 10% glycerol and 1 mM DTT) supplemented with protease inhibitors (Roche, cat. #11836153001). The resulting lysate was incubated with anti-FLAG M2 magnetic beads (Sigma, cat. #8823) at 4 °C for 2 h, and the beads were washed with lysis buffer five times, and Sirt7 buffer (50 mM Tris-HCl (pH 8.0), 100 mM NaCl, 5 mM DTT, and 20% glycerol) three times, sequentially. The immobilized proteins were eluted with 3 × FLAG peptide, and ultrafiltration was carried out using using an Amicon Ultra-0.5 10 K device (Merck Millipore, cat. #UFC501024) to concentrate the eluted proteins.

### Pull-down assay by FLAG-Sirt7

To a 150 µL of solution of nucleosomes (0.05 µM) and FLAG-Sirt7 (WT, DM, or CA) (0.17 ng/µL) in buffer A (20 mM Tris (pH 8.0), 100 mM NaCl, 5 mM MgCl_2_, 1 mM DTT, 2.5% glycerol, 0.01% Triton X-100), 5 µL of anti-FLAG M2 magnetic beads (Sigma, #M8823) in 50 µL of buffer A was added. After incubation at 4 °C for 90 min, supernatant was removed using a magnetic rack. The beads were washed with 1 mL of buffer A twice, and boiled with SDS loading buffer. The resulting supernatant was analyzed by Oriole stain and western blotting by anti-histone H3 antibody (Abcam, #1791).

### Data availability

The datasets generated during and/or analyzed during the current study are available from the corresponding author on reasonable request.

## Electronic supplementary material

Supplementary Information
