# Supplementary material for: LC–MS/MS-based quantitative study of the acyl group- and site-selectivity of human sirtuins to acylated nucleosomes
Source: Sci Rep. 2018 Feb 8;8:2656. doi: 10.1038/s41598-018-21060-2 (PMC5805777; doi:10.1038/s41598-018-21060-2)
Supplement: Supplementary file 1 — Supplementary Information [file 41598_2018_21060_MOESM1_ESM.pdf]

Supplementary Information for:

# *LC–MS/MS-based quantitative study of the acyl group- and site-selectivity of human sirtuins to acylated nucleosomes*

*Kana Tanabe[1,2], Jiaan Liu[1], Daiki Kato[3], Hitoshi Kurumizaka[3], Kenzo Yamatsugu[1,2], Motomu Kanai[1,2], & Shigehiro A. Kawashima[1,2]*

*[1]Graduate School of Pharmaceutical Sciences, The University of Tokyo, 7-3-1 Hongo, Bunkyo-ku, Tokyo 113-0033, Japan, [2]JST-ERATO, [3]Laboratory of Structural Biology, Graduate School of Advanced Science and Engineering, Waseda University, 2-2 Wakamatsu-cho, Shinjuku-ku, Tokyo 162-8480, Japan*

Contents:

1. Synthesis of **3Bu8R** (**2**)
2. Supplementary Tables and Figures

## 1. Synthesis of 3Bu8R (2)

### General

$^{1}$H NMR spectra were recorded on JEOL ECX500 (500 MHz for $^{1}$H NMR and 125 MHz for $^{13}$C NMR) spectrometer. Chemical shifts were reported in ppm on the δ scale relative to residual $CHCl_3$ (δ = 7.24 for $^{1}$H NMR and δ = 77.0 for $^{13}$C NMR) as an internal reference. Column chromatographies were performed with Kanto silica gel 60 (40–50 mesh). Analytical HPLC was conducted by using a JASCO HPLC system equipped with a UV–2075 spectrometer, PU–2080 pumps, a DG–2080–54 degasser, an MX–2080–32 mixer. Preparative HPLC was conducted by using a JASCO HPLC system equipped with a UV–2075 spectrometer, PU–2086 pumps, a DG–2080–53 degasser, an MX–2080–32 mixer. ESI–MS spectra were measured on a Shimadzu LCMS-2020 (for LRMS), and a JEOL JMS-T100LC AccuTOF spectrometer (for HRMS). MALDI–TOF MS was obtained with a Shimadzu Biotech Axima ToF$^{2}$ spectrometer. Reagents were purchased from Aldrich, Tokyo Chemical Industry Co., Ltd. (TCI), or Wako Pure Chemical Industries, Ltd., and used as received.

### Analytical HPLC

Analytical HPLC was performed using YMC–Triart C18 (4.6 mm I.D. × 150 mm) column using a gradient of acetonitrile in 0.1% aqueous TFA at 40 °C with a flow rate of 1 mL/min. The eluent was monitored by absorbance at 230 nm.
Gradient: 2% acetonitrile for 5 min, followed by a linear gradient of 2-98% acetonitrile over 37 min.

### Preparative HPLC

Preparative HPLC was performed using YMC–Triart C18 (10 mm I.D. × 250 mm) column using a gradient of acetonitrile in 0.1% aqueous TFA at 40 °C with a flow rate of 3 mL/min. The eluent was monitored by absorbance at 230 nm.
Gradient: 10% acetonitrile for 10 min, followed by a linear gradient of 10-90% acetonitrile over 50 min.

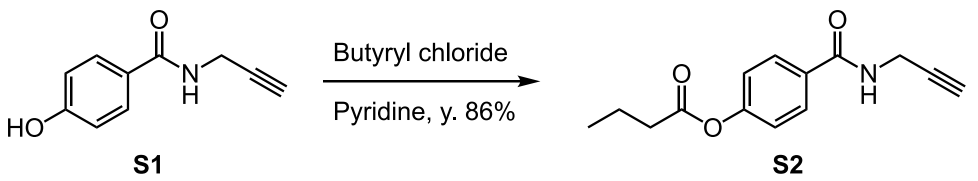


**4–(Prop–2–yn–1–ylcarbamoyl)phenyl butyrate (S2):**

To a stirred solution of **S1**[1] (17.5 mg, 0.0998 mmol) in dried pyridine (1.00 mL), butyryl chloride (15.1 $\mu$L, 0.146 mmol) was added dropwise under ice–water bath, then the mixture was warmed to room temperature and stirred for 2 h. Volatiles were removed under reduced pressure, and the residue was purified with silica gel column chromatography (EtOAc/Hexane = 1/3) to afford **S2** (21.2 mg, 0.0864 mmol, y. 86%) as white solids. $^{1}$H NMR ($CDCl_3$, 500 MHz) δ 7.76 (d, *J* = 8.6 Hz, 2H), 7.09 (d, *J* = 8.6 Hz, 2H), 6.61 (brs, 1H), 4.17 (dd, *J* = 2.5, 5.1 Hz, 2H), 2.52 (t, *J* = 7.4 Hz, 2H), 2.23 (t, *J* = 2.5 Hz, 1H), 1.79–1.71 (m, 2H), 1.01 (t, *J* = 6.8 Hz, 1H); $^{13}$C NMR ($CDCl_3$, 125 MHz) δ 171.7, 166.2, 153.2, 131.1, 128.5, 121.7, 79.4, 71.7, 36.1, 29.6, 18.2, 13.5; ESI–MS *m*/*z* 268 $[M+Na]^+$; ESI-HRMS: *m*/*z* calcd for $C_{14}H_{15}NNaO_3$ $[M+Na]^+$: 268.0944. Found: 268.0946.

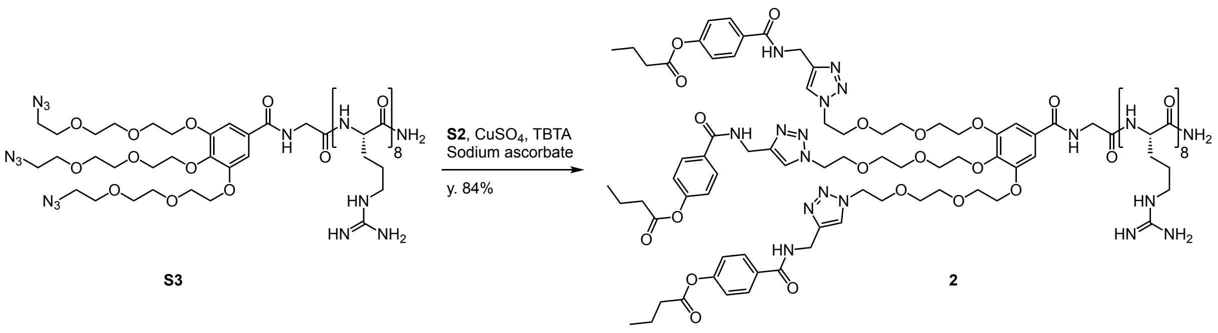


**3Bu-8R (2):**

To a stirred solution of **S3**[2] (3.39 mg, 0.00118 mmol calculated as an 8TFA salt) in water–*t*BuOH (1 : 1, 1.18 mL), alkyne **S2** solution in $CH_3CN$ (160 mM, 0.059 mL, 0.00944 mmol), Cu–TBTA (Tris[(1-benzyl-1*H*-1,2,3-triazol-4-yl)methyl]amine) solution in water–*t*BuOH ($CuSO_4$ : TBTA = 1 : 1, water : *t*BuOH = 1 : 1, 7.69 mM, 0.153 mL, 0.00118 mmol), and sodium ascorbate aqueous solution (50 mM, 0.118 mL, 0.0059 mmol) were added, and the mixture was stirred at 4 °C for 1 h. The mixture was directly purified by preparative HPLC to afford **2** (3.59 mg, 0.000998 mmol calculated as an 8TFA salt, y. 84 %) as white solids after lyophilization.

MALDI–TOF MS (CHCA) *m*/*z* calcd 2682.45 $[M+H]^+$, Found: 2682.03. Retention time: 23.6 min.

## 2. Supplementary Tables and Figures

**Table S1.** List of the purchased sirtuins

| | manufacturer | cat No. | expression system | tag | Mw |
|---|---|---|---|---|---|
| **Sirt1** | ActiveMotif | #31340 | *E. coli* | FLAG | ~120 kDa |
| **Sirt2** | MyBiosource | #MBS203851 | *E. coli* | His | 41.7 kDa |
| **Sirt3** | Cyclex | #CY-E1153 | *E. coli* | GST | 56 kDa |
| **Sirt5** | Cyclex | #CY-E1155 | *E. coli* | GST | 60 kDa |
| **Sirt6** | Cyclex | #CY-E1156 | *E. coli* | GST | 64 kDa |
| **Sirt7** | Sigma | #SRP5274 | baculovirus infected Sf9 cells | His | 46 kDa |

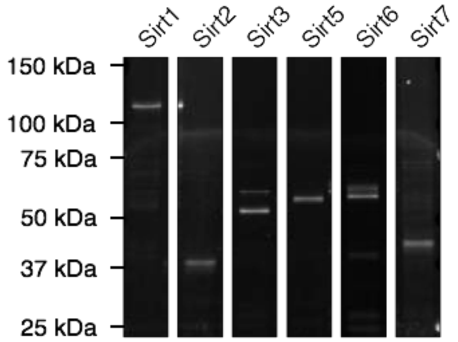


**Figure S1.** SDS-PAGE analyses of the purchased sirtuins, detected by Oriole stain.

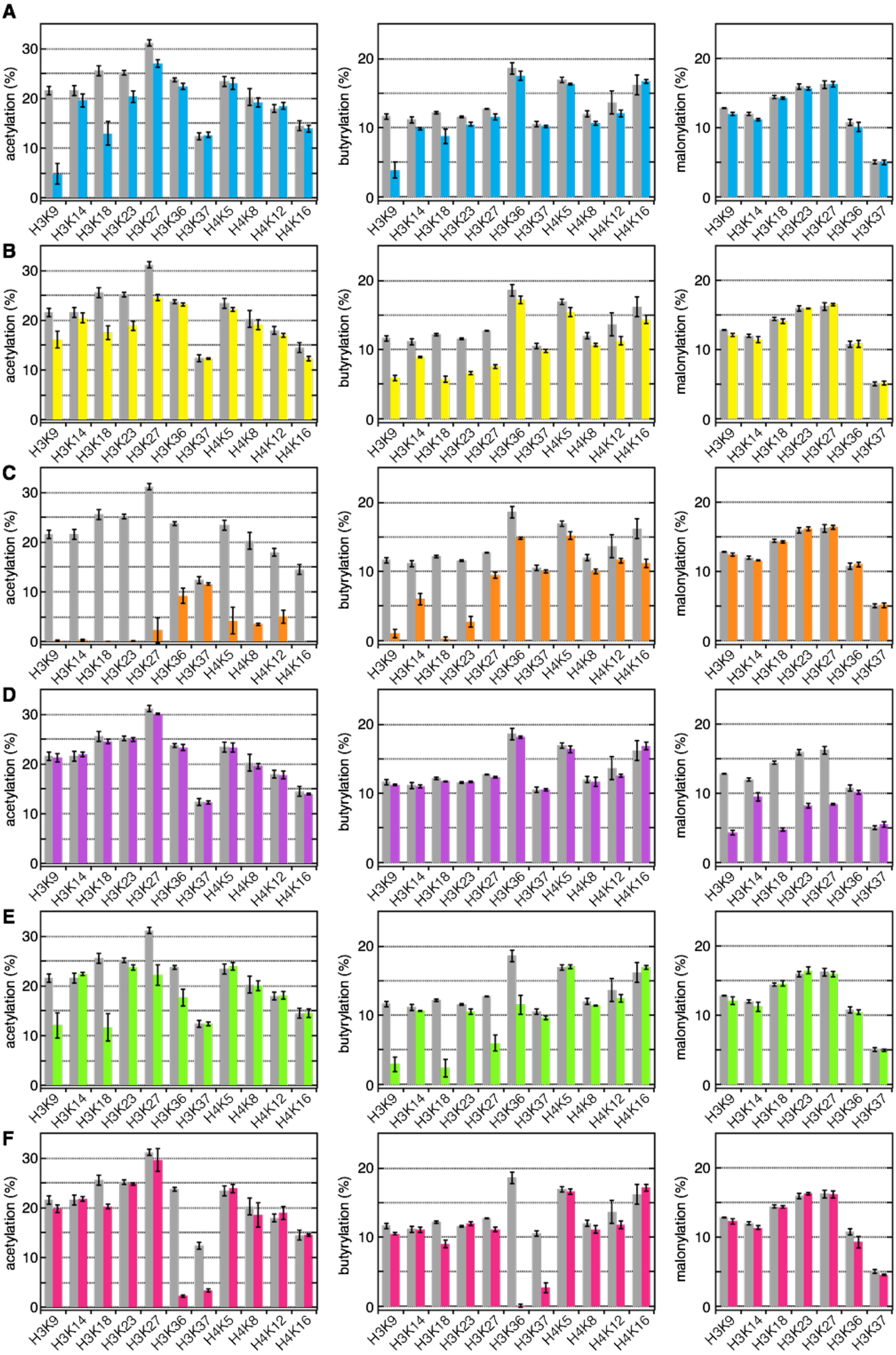
A
B
C
D
E
F
acetylation (%)
butyrylation (%)
malonylation (%)
H3K9
H3K14
H3K18
H3K23
H3K27
H3K36
H3K37
H4K5
H4K8
H4K12
H4K16

**Figure S2.** The stoichiometry of acylations on H3 and H4 tails on deacetylation (left), debutyrylation (right), and demalonylation (right) assays. Gray columns indicate control samples without HDAC, and colored columns are sirtuin-treated samples in all the charts. (A) Results for HDAC assay of Sirt1 (24 ng/$\mu$L). (B) Results for HDAC assay of Sirt2 (24 ng/$\mu$L). (C) Results for HDAC assay of Sirt3 (12 ng/$\mu$L). (D) Results for HDAC assay of Sirt5 (2 ng/$\mu$L). (E) Results for HDAC assay of Sirt6 (12 ng/$\mu$L). (F) Results for HDAC assay of Sirt7 (12 ng/$\mu$L). The average and SD (error bars) obtained from $n$ = 3 independent HDAC assays are indicated, where measurements were conducted in duplicate for each HDAC assay.

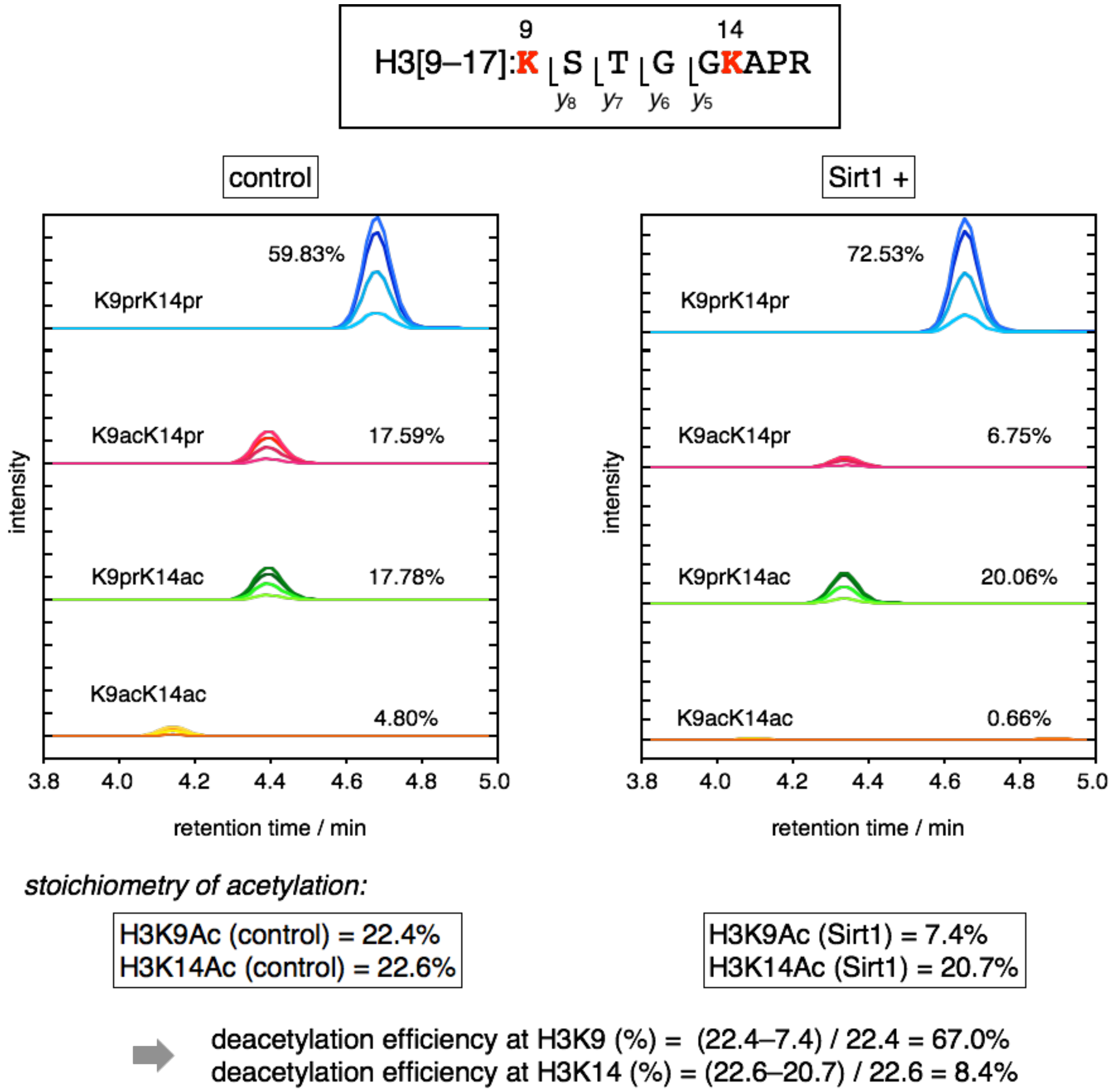


**Figure S3**. An example of calculation for deacetylation efficiency. Acetylated nucleosome was treated with (right) or without (left) Sirt1 (24 ng/$\mu$L) at 30 ºC for 3 h. Extracted ion chromatograms of tryptic peptides H3[9–17], with precursor ions of 507.29 (K9prK14pr), 500.28 (K9acK14pr and K9prK14ac), and 493.27 (K9acK14ac) and the following four fragment ions ($y^8$, $y^7$, $y^6$, and $y^5$) with a tolerance of ± 0.2 Da: K9prK14pr and K9acK14pr, 829.45 ($y^8$), 742.42 ($y^7$), 641.37 ($y^6$), and 584.35($y^5$); K9prK14ac and K9acK14ac, 815.45 ($y^8$), 728.40 ($y^7$), 627.36 ($y^6$), and 570.34 ($y^5$). The peak area of each extracted ion chromatogram was calculated with PeakView software. The stoichiometry of acetylation was calculated with the peak area by the equation in Figure 1D. The resulting deacetylation efficiency was obtained with the equation in Figure 1E.

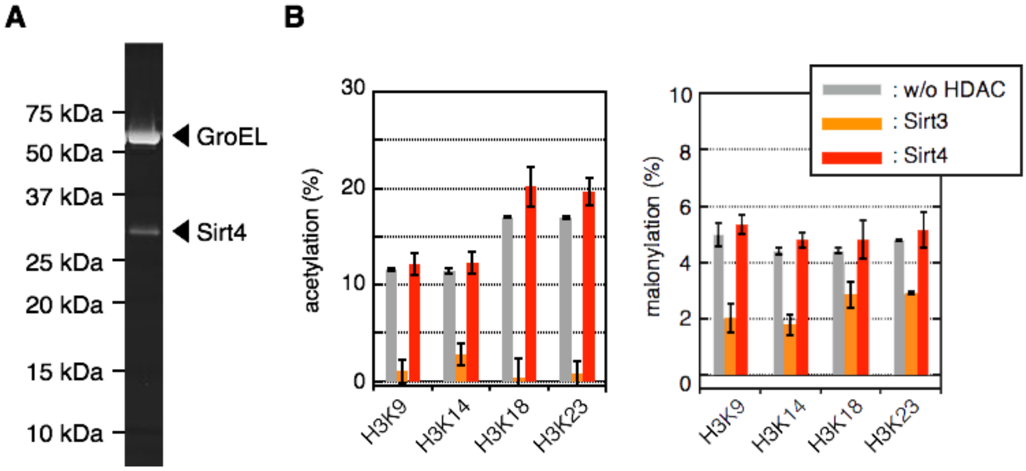


**Figure S4.** Deacetylation by Sirt4. (A) Oriole stain of the purified recombinant Sirt4. (B) The stoichiometry of acetylation on H3 tails on deacetylation or demalonylation assays. Gray columns indicate samples without any HDAC, orange columns are sirt3-treated sample (1 ng/$\mu$L) as a positive control, red columns are Sirt4-treated sample (3 ng/$\mu$L). Error bars indicate upper and lower limits of two independent measurements.

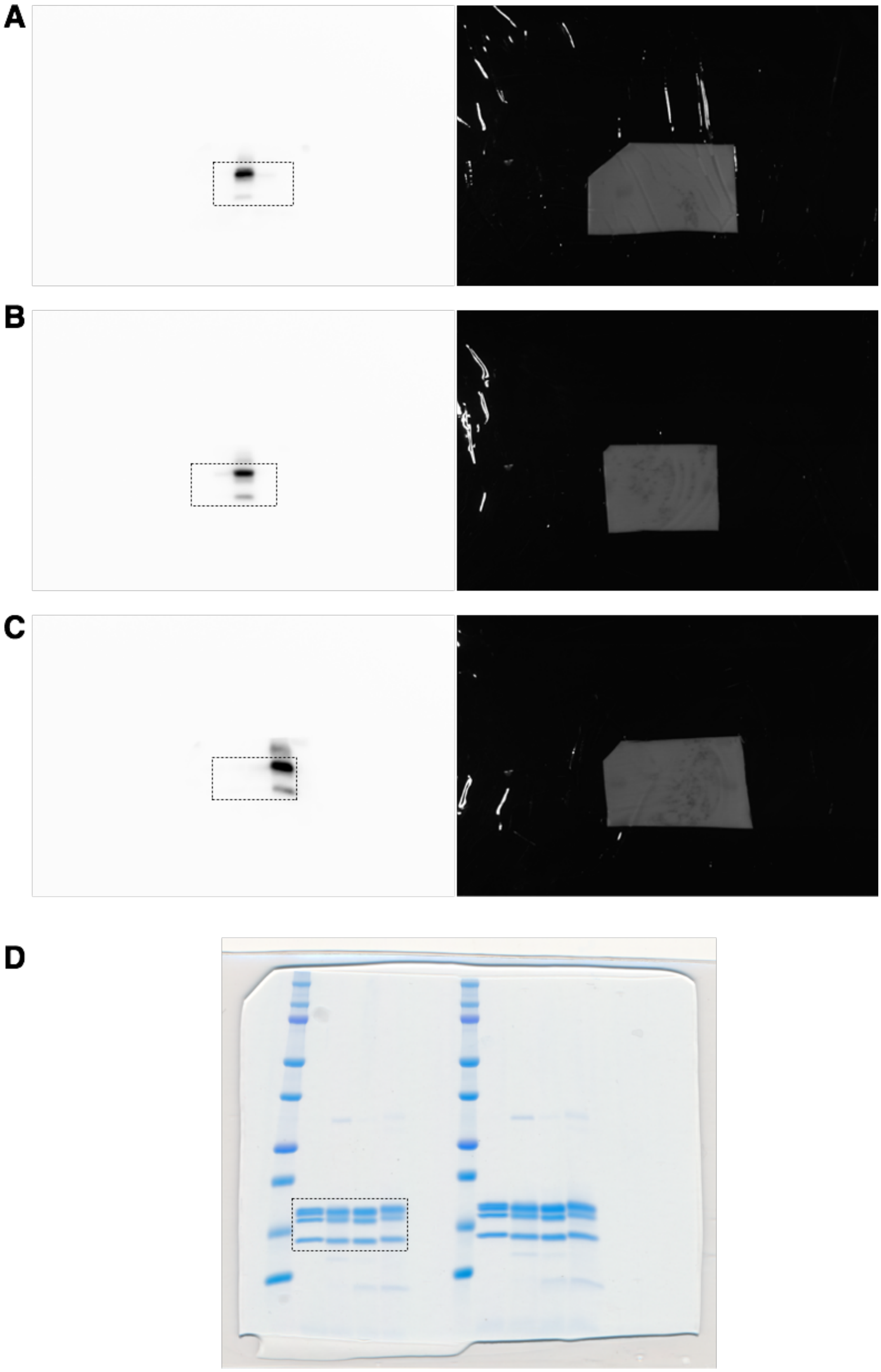


**Figure S5.** Uncropped images for Figure 1B. Membranes detected by antibodies of (A) anti-LysAc, (B) anti-LysBu, and (C) anti-LysMal, and (D) the gel stained by Coomassie brilliant blue.

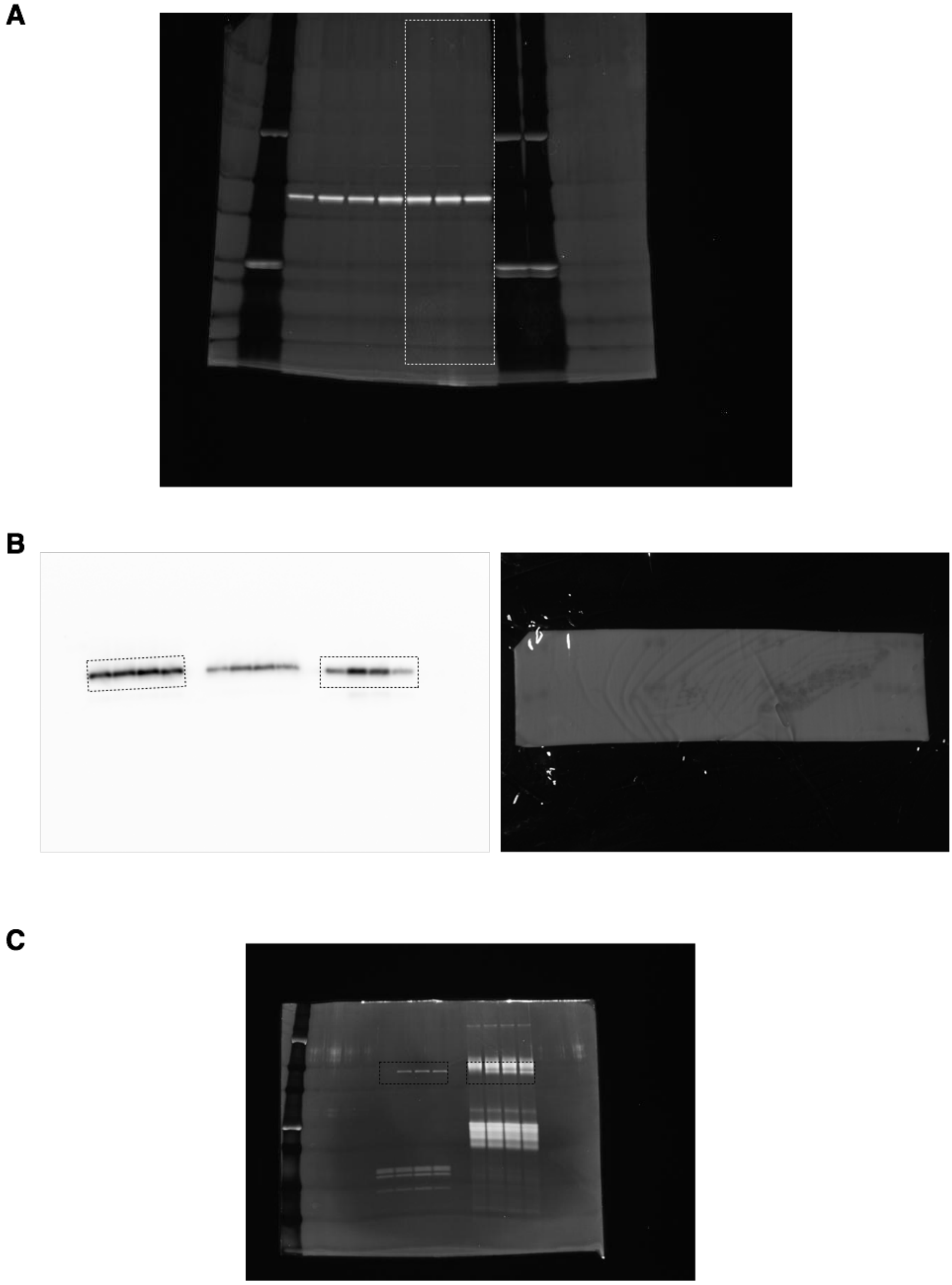


**Figure S6.** Uncropped images for Figures 5B and 5G. (A) Oriole stain in Figure 5B. (B) Immunoblot in Figure 5G. The left four lanes indicate input samples, and the right four lanes show IP samples. (C) Oriole stain for input (left four signals) and IP (right four signals) samples in Figure 5G.

**Table S2.** LC–MS/MS parameters for detection and analysis of tryptic peptides from histones H3, H4, and H2A.

| peptide | sequence | precuesor ion ($m/z$)[a] | fragment ions | collision energy (V) | retention time (min) |
|---|---|---|---|---|---|
| H3[3–8] | TKQTAR | 380.72 (1Pr) | $y_2, y_3, y_4, y_5$ | 35 | 2.2–3.2 |
| | | 387.72 (1Bu) | | | |
| H3[9–17] | KSTGGKAPR | 507.29 (2Pr) | $y_5, y_6, y_7, y_8$ | 35 | 2.8–5.0 |
| | | 500.28 (1Pr 1Ac) | | | |
| | | 493.27 (2Ac) | | | |
| | | 514.30 (1Pr 1Bu) | | | |
| | | 521.31 (2Bu) | | | |
| | | 537.26 (1Pr 1Mal) | | | |
| | | 522.28 (2Mal) | | | |
| H3[18–26] | KQLATKAAR | 549.84 (2Pr) | $y_5, y_6, y_7, y_8$ | 35 | 3.8–6.0 |
| | | 542.83 (1Pr 1Ac) | | | |
| | | 535.82 (2Ac) | | | |
| | | 556.84 (1Pr 1Bu) | | | |
| | | 563.85 (2Bu) | | | |
| | | 564.82 (1Pr 1Mal) | | | |
| | | 579.81 (2Mal) | | | |
| H3[27–40] | KSAPATGGVKKPHR | 534.64 (3Pr) | $y_5, y_6, y_7, y_8; y_4$ | 35 | 3.8–6.5 |
| | | 529.97 (2Pr 1Ac) | | | |
| | | 525.30 (1Pr 2Ac) | | | |
| | | 520.63 (3Ac) | | | |
| | | 539.31 (1Bu 2Pr) | | | |
| | | 543.99 (2Bu 2Pr) | | | |
| | | 548.66 (3Bu) | | | |
| | | 544.64 (2Pr 1Mal) | | | |
| | | 554.63 (1Pr 2Mal) | | | |
| | | 564.62 (3Mal) | | | |
| H3[54–63] | YQKSTELLIR | 653.87 (1Pr) | $b_3, y_6, y_7, y_8$ | 35 | 5.9–6.9 |
| | | 660.8 (1Bu) | | | |
| H3[64–69] | KLPFQR | 422.76 (1Pr) | $y_2, y_3, y_4, y_5$ | 35 | 5.3–7.3 |
| | | 429.76 (1Bu) | | | |

| | | | | | |
|---|---|---|---|---|---|
| H3[73–83] | EIAQDFKTDLR | 696.36 (1Pr) | $y_5, y_6, y_7, y_8$ | 35 | 5.9–6.9 |
| | | 703.37 (1Bu) | | | |
| H3[117–128] | VTIMPKDIQLAR | 720.72 (1Pr) | $y_8, y_9, y_{10}, b_3$ | 35 | 5.8–6.8 |
| | | 727.92 (1Bu) | | | |
| H4[4–17] | GKGGKGLGKGGAKR | 747.94 (4Pr) | $y_3, y_4, y_5; b_2, b_3,$ | 45 | 4.0–6.0 |
| | | 740.93 (3Pr 1Ac) | $b_4; y_7, y_8, y_9$ | | |
| | | 733.93 (2Pr 2Ac) | | | |
| | | 726.92 (1Pr 3Ac) | | | |
| | | 719.91 (4Ac) | | | |
| | | 754.95 (3Pr 1Bu) | | | |
| | | 761.96 (2Pr 2Bu) | | | |
| | | 768.96 (1Pr 3Bu) | | | |
| | | 775.97 (4Bu) | | | |
| H4[20–23] | KVLR | 286.20 (1Pr) | $y_1, y_2, y_3$ | 35 | 3.5–5.0 |
| | | 293.21 (1Bu) | | | |
| H4[24–35] | DNIQGITKPAIR | 691.39 (1Pr) | $y_6, y_7, y_8, y_9$ | 45 | 5.6–6.6 |
| | | 698.40 (1Bu) | | | |
| H4[41–45] | GGVKR | 286.68 (1Pr) | $y_1, y_2, y_3$ | 35 | 2.5–4.0 |
| | | 293.69 (1Bu) | | | |
| H4[56–67] | GVLKVFLENVIR | 721.94 (1Pr) | $y_6, y_7, y_8, y_9$ | 40 | 7.6–9.0 |
| | | 728.95 (1Bu) | | | |
| H4[68–78] | DAVTYTEHAKR | 673.84 (1Pr) | $y_5, y_6, y_7, y_8$ | 60 | 4.0–5.0 |
| | | 680.85 (1Bu) | | | |
| H4[79–92] | KTVTAMDVVYALKR | 853.98 (2Pr) | $y_8, y_9, y_{10}, y_{11}$ | 40 | 7.0–8.5 |
| | | 860.99 (1Pr 1Bu) | | | |
| | | 867.99 (2Bu) | | | |
| H2A[4–11] | GKQGGKAR | 457.26 (2Pr) | $y_4, y_5, y_6$ | 35 | 3.0–4.5 |
| | | 464.27 (1Pr 1Bu) | | | |
| | | 471.28 (2Bu) | | | |
| H2A[12–17] | AKAKTR | 393.75 (2Pr) | $b_2, y_3, y_4$ | 35 | 3.0–4.5 |
| | | 400.75 (1Pr 1Bu) | | | |
| | | 407.76 (2Bu) | | | |
| H2A[36–42] | KGNYSER | 455.22 (1Pr) | $y_3, y_4, y_5, y_6$ | 35 | 3.0–4.5 |
| | | 462.23 (1Bu) | | | |
| H2A[72–77] | DNKKTR | 437.24 (2Pr) | $y_3, b_3$ | 35 | 3.0–4.5 |

| |
|---|
| 444.25 (1Pr 1Bu) |
| 451.26 (2Bu) |

[a] *n*Ac *m*Pr in parenthesis means that *n* lysines are acetylated out of all the (*n*+*m*) lysines on the corresponding peptide.

**Table S3.** Correction parameters of ionization efficiency for malonylated peptides and propionylated peptides.

| peptide | sequence | fragment ions | correction parameters[a] |
|---|---|---|---|
| H3[9–17]K9Mal | K(Mal)STGGK(Pr)APR | $y_3$ | 1.49 |
| | | $y_4$ | 1.33 |
| | | $y_5$ | 1.22 |
| | | $y_6$ | 1.14 |
| H3[9–17]K14Mal | KSTGGK(Mal)APR | $y_3$ | 1.12 |
| | | $y_4$ | 1.10 |
| | | $y_5$ | 1.07 |
| | | $y_6$ | 1.11 |
| H3[9–17]K9MalK14Mal | K(Mal)STGGK(Mal)APR | $y_3$ | 1.30 |
| | | $y_4$ | 1.19 |
| | | $y_5$ | 1.08 |
| | | $y_6$ | 1.04 |
| H3[18–26]K18Mal | K(Mal)QLATKAAR | $y_3$ | 1.03 |
| | | $y_4$ | 1.10 |
| | | $y_5$ | 1.16 |
| | | $y_6$ | 1.38 |
| H3[18–26]K23Mal | KQLATK(Mal)AAR | $y_3$ | 1.10 |
| | | $y_4$ | 1.09 |
| | | $y_5$ | 1.09 |
| | | $y_6$ | 1.11 |
| H3[18–26]K18MalK23Mal | K(Mal)QLATK(Mal)AAR | $y_3$ | 1.23 |
| | | $y_4$ | 1.29 |
| | | $y_5$ | 1.35 |
| | | $y_6$ | 1.61 |

[a] correction parameters were calculated as (peak area of authentic propionylated peptide)/(peak area of authentic malonylated peptide)

## References

1) Amir, R. J., Danieli, E. & Shabat, D. Receiver-amplifier, self-immolative dendritic dievice. *Chem. Eur. J.* **13**, 812-821 (2007).

2) Ishiguro, T. *et al*. Synthetic chromatin acylation by an artificial catalyst system. *Chem* **2**, 840-859 (2017).
